# Evidence of Porcine Circovirus Type 2 (PCV2) Genetic Shift from PCV2b to PCV2d Genotype in Sardinia, Italy

**DOI:** 10.3390/v15112157

**Published:** 2023-10-26

**Authors:** Silvia Dei Giudici, Lorena Mura, Piero Bonelli, Salwa Hawko, Pier Paolo Angioi, Anna Maria Sechi, Stefano Denti, Antonella Sulas, Giovanni Pietro Burrai, Maria Paola Madrau, Elisabetta Antuofermo, Annalisa Oggiano

**Affiliations:** 1 Department of Animal Health, Istituto Zooprofilattico Sperimentale della Sardegna, 07100 Sassari, Italy; lorena.mura@izs-sardegna.it (L.M.); piero.bonelli@izs-sardegna.it (P.B.); pierpaolo.angioi@izs-sardegna.it (P.P.A.); annamaria.sechi@izs-sardegna.it (A.M.S.); stefano.denti@izs-sardegna.it (S.D.); antonella.sulas@izs-sardegna.it (A.S.); mariapaola.madrau@izs-sardegna.it (M.P.M.); annalisa.oggiano@izs-sardegna.it (A.O.); 2 Department of Veterinary Medicine, University of Sassari, 07100 Sassari, Italy; salwa.hawko@hotmail.com (S.H.); gburrai@uniss.it (G.P.B.); eantuofermo@uniss.it (E.A.)

**Keywords:** Porcine Circovirus type 2, phylogenetic analysis, genetic shift

## Abstract

Porcine Circovirus type 2 (PCV2) is the etiological agent of a disease syndrome named Porcine Circovirus disease (PCVD), representing an important threat for the pig industry. The increasing international trade of live animals and the development of intensive pig farming seem to have sustained the spreading of PCVD on a global scale. Recent classification criteria allowed the identification of nine different PCV2 genotypes (PCV2a–i). PCV2a was the first genotype detected with the highest frequency from the late 1990s to 2000, which was then superseded by PCV2b (first genotype shift). An ongoing genotype shift is now determining increasing prevalence rates of PCV2d, in replacement of PCV2b. In Italy, a complete genotype replacement was not evidenced yet. The present study was carried out on 369 samples originating from domestic pigs, free-ranging pigs, and wild boars collected in Sardinia between 2020 and 2022, with the aim to update the last survey performed on samples collected during 2009–2013. Fifty-seven complete ORF2 sequences were obtained, and the phylogenetic and network analyses evidenced that 56 out of 57 strains belong to the PCV2d genotype and only one strain to PCV2b, thus showing the occurrence of a genotype shift from PCV2b to PCV2d in Sardinia.

## 1. Introduction

Porcine circoviruses (PCV) are the smallest single-stranded (ss) DNA viruses belonging to the family Circoviridae, genus *Circovirus*. Circoviruses have a circular genome of 1.7–2.0 kb in length and icosahedral virions. Until now, four species in the genus *Circovirus*, causing infection in pigs, were identified: PCV1, PCV2, PCV3, and PCV4. PCV1 was first detected in 1974 [1] as a PK-15-cell culture contaminant, and it is considered non-pathogenic for swine [2]. PCV2, first isolated in 1998, is described as the etiological agent of a disease syndrome nowadays named Porcine Circovirus Diseases (PCVD) [3,4], but originally described as Postweaning Multisystemic Wasting Syndrome (PMWS) [5,6]. PCVD grouped several pathological conditions with the involvement of multiple organ systems, determining an important economic impact on the pig industry worldwide [7,8]. In 2017, PCV3 was first identified in the USA [9] and subsequently found in Asia and Europe [10,11,12,13], whereas, more recently, PCV4 (2019) was detected in China and South Korea [14,15]. Although PCV3 and PCV4 were detected in association with various clinical conditions in pigs, often in co-infections with other viral pathogens, their pathogenicity is still controversial [10,14,16,17].

PCV2 is now considered a ubiquitous virus representing a major threat to the pig industry. The increase in international trade of live animals and the development of intensive pig farming seem to have sustained the epidemic proportions of PCVD on a global scale [8,18]. Wild boars are also susceptible to PCV2 infection even though they show lower viral titers than domestic pigs and seldom display the clinical condition [19,20]. A high prevalence of PCV2 in wild boars was reported in several European countries [21,22], confirming the actual role of wild boars as reservoirs of PCV2 in nature. PCV2 has also been isolated from other animal species (ruminants, rodents, canines, insects, and humans) suggesting the presence of many types of reservoirs and the possible cross-species transmission of PCV2 [23].

The genome of PCV2 hypothetically comprises at least 11 identified open reading frames (ORF), but only 6 of them have been described to encode for different structural and non-structural proteins [24]. ORF1 encodes for two proteins (Rep and Rep’) associated with the replication process of the virus, whereas ORF2, also called the *cap* gene, encodes for the viral capsid structural protein [25]. Of note, ORF2 is a very important component in the virus/host attachment and is used for PCV2 genotyping [26]. The remaining four ORFs (ORF3 to ORF6), coding for non-structural proteins, are associated with cell apoptosis and interaction with other proteins, leading to replication enhancement of the virus and its survival [27,28,29].

Similar to RNA viruses, PCV2 is characterized, among all ssDNA viruses, by the highest variability of nucleotide substitution (10^−3^–10^−4^ substitution/site/year) [30]. A study conducted by Franzo and coauthors in 2018 [31] proposed updated classification criteria allowing the identification of eight different PCV2 genotypes (PCV2a-h). Using the same criteria, a new genotype, PCV-2i, was proposed by Wang et al. [32].

Over the past decades, there has been the emergence of new PCV2 genetic variants, which is the evolution of the virus determined by the combined effect of its high mutation rate and the pig industry process [33]. Recently, phylodynamic studies reported that PCV2 genotypes appear in periodic waves, spread depending on the international swine trade, and are then replaced by emerging PCV2 genotypes [34]. PCV2a was the first genotype detected with the highest frequency from the late 1990s to 2000, which was then superseded by PCV2b (first genotype shift) [7,18]. An ongoing genotype shift is now determining increasing prevalence rates of the emerging PCV2d, in replacement of PCV2b, in Europe, Asia, and America [8,19,25,35,36,37]. Our previous study on PCV2 performed using sequences collected in 2009–2013 showed the prevalent circulation in Sardinia of the PCV2b genotype, and dated back to 2011, the introduction of PCV2d in the Italian island [38]. Since the first detection of PCV2d in Italy in 2010 [39], a progressive increase in its prevalence has been reported in this country even though an actual genotype replacement cannot be evidenced [19].

The rise in PCV2d prevalence worldwide highlights the necessity of further investigation into PCV2 molecular epidemiology. The present study was carried out on samples originating from domestic and wild pig populations to detect genetic variants circulating in Sardinia. The occurrence of the genotype shift of Sardinian PCV2 from PCV2b to PCV2d was also evaluated.

## 2. Materials and Methods

### 2.1. Samples

In this study, 369 samples (133 blood sera and 236 tissues) from domestic and wild suids were collected in the years 2020–2022 and analyzed at the Diagnostic Virology Laboratory of Istituto Zooprofilattico Sperimentale (IZS) of Sardinia:

- 204 samples (89 blood sera and 115 tissue samples) from domestic pigs throughout the Sardinian regional territory;
- 102 samples (44 blood sera and 58 spleens) from hunted wild boars collected during the 2021/2022 hunting season in north Sardinia (Sassari province);
- 63 spleen samples obtained from feral free-ranging pigs culled between 2021 and 2022 in central Sardinia (Nuoro province) during the depopulation action of the Sardinian African Swine Fever Virus (ASFV) eradication plan.

Free-ranging pigs and wild boars were not vaccinated against PCV. No data were available about the immune status of domestic pigs sampled in this study.

### 2.2. Detection of PCV2

Viral DNA was extracted from blood sera or tissue samples using a MagMax Core kit and MagMax96 extractor (Thermo Fisher, Waltham, MA, USA) following the manufacturer’s instructions. DNA samples were stored at –20 °C until further analyses. All the samples were screened for PCV2 by a real-time PCR assay, as previously described [40]. Samples with a Ct value of less than 40 were considered positive.

Chi-squared test (Χ^2^) was used to analyze the differences in PCV2 detection rate between domestic pigs, free-ranging pigs, and wild boars using SPSS software v.21. *p* values < 0.05 were regarded as statistically significant.

### 2.3. ORF2 Sequencing

Fifty-seven PCV2 positive samples (domestic pigs, *n* = 26; wild boars, *n* = 25; free-ranging pigs, *n* = 6) were randomly selected, and the complete ORF2 nucleotide sequences were amplified as previously described [41]. Sanger sequencing was performed on both strands on an ABI-PRISM 3500 Genetic Analyzer (Applied Biosystems, Waltham, MA, USA) with a DNA sequencing kit (dRhodamine Terminator Cycle Sequencing Ready Reaction; Applied Biosystems, Waltham, MA, USA). The sequences were assembled, edited, aligned, and translated using BioEdit software, version 7.0.0 [42].

### 2.4. Phylogenetic Analyses

Two ORF2 data sets were built. The first was used for genotyping (*n* = 81) and was composed of 57 sequences obtained in the present study and 24 reference sequences representative of the PCV2a–h genotypes [31,37]. The second data set (*n* = 192) was compiled from the sequences obtained in the present study (*n* = 57), PCV2b and PCV2d reference strains (*n* = 6) [31], all available complete ORF2 sequences from Italy (*n* = 48), GenBank sequences from different countries with the highest similarity with Sardinian strains (*n* = 41), and sequences from our previous work (*n* = 40) [38].

The data sets were checked for recombination by RDP, GENECONV, MaxChi, and 3Seq methods in the RDP4 software [38,43]. The evolutionary model that best fitted the data for the data sets was selected using JmodelTest v.2.1.7 [44]. The phylogenetic signal of all the data sets was subjected to the likelihood mapping analysis with 10,000 random quartets in the TreePuzzle software v. 5.2 as already described [38]. Phylogenies were reconstructed using both data sets in MEGA 7 [45] via the maximum likelihood and GTR+G+I model of nucleotide substitution. Statistical support for specific clades was obtained via 1000 bootstrap replicates.

Haplotype network analysis was performed on the second dataset, excluding the singleton haplotypes not obtained in this study (*n* = 138; Table S2). Haplotypes were identified using DnaSP version 5 [46], and TCS networks were calculated using PopART software v 1.7 [47].

### 2.5. Epitope Prediction and Amino Acid Mutation Analysis

Fifty-seven *cap* amino acid sequences obtained in this study were aligned with PCV2b and PCV2d reference strains. Epitope prediction to evaluate the B cell and T cell epitopes of the *cap* protein from our PCV2d strains compared to MF314285 CBI090 PCV-2d reference strain was performed using the online servers ABCpred (https://webs.iiitd.edu.in/raghava/abcpred/ABC_submission.html accessed on 16 October 2023) and NetCTL-1.2 (https://services.healthtech.dtu.dk/service.php?NetCTL-1.2, accessed on 16 October 2023), respectively.

Amino acid mutation analysis was performed on the characteristic amino acid replacements found in this study using Missence3D (http://missense3d.bc.ic.ac.uk/~missense3d/, accessed on 16 October 2023).

## 3. Results

The PCV2 viral genome was detected in 204 out of 369 samples (55.28%, Table 1). The overall infection rate, calculated including blood serum and tissue positive samples, was 36.27% in domestic pigs, 87.3% in free-ranging pigs, and 73.53% in wild boars (Table 1).

Significant differences were evidenced in the infection rate of domestic pigs compared to wild boars (Χ^2^ = 36.30, *p* = 0.00001) and free-ranging pigs (Χ^2^ = 48.17, *p* = 0.00001); instead, no significant difference between wild boars and free-ranging pigs was detected (Χ^2^ = 3.63, *p* = 0.057). In Table 1, the results related to the different matrices analyzed are reported. PCV2 detection in blood sera was lower in domestic pigs than in wild boars (Χ^2^ = 12.95, *p* = 0.00035). The infection rate in tissue samples was significantly lower in domestic pigs than in wild boars (Χ^2^ = 26.48, *p* = 0.00001) and free-ranging pigs (Χ^2^ = 32.31, *p* = 0.00001), and no difference between free-ranging pigs and wild boars (Χ^2^ = 0.20, *p* = 0.66) was observed.

The sequences of the complete ORF2 of 57 Sardinian samples were deposited in GenBank (OR519918-OR519974), and detailed information is presented in Tables S1 and S2. The analysis of the electropherogram showed the presence of a few double peaks in four sequences (389, 462, 37203, 80140); for each sample, only the major variant was deposited in GenBank and utilized in this study.

The *cap* gene sequences were 702 or 705 bp long and showed 92.3–100% similarity among them. Twenty-eight different haplotypes were evidenced.

**Figure 1:**
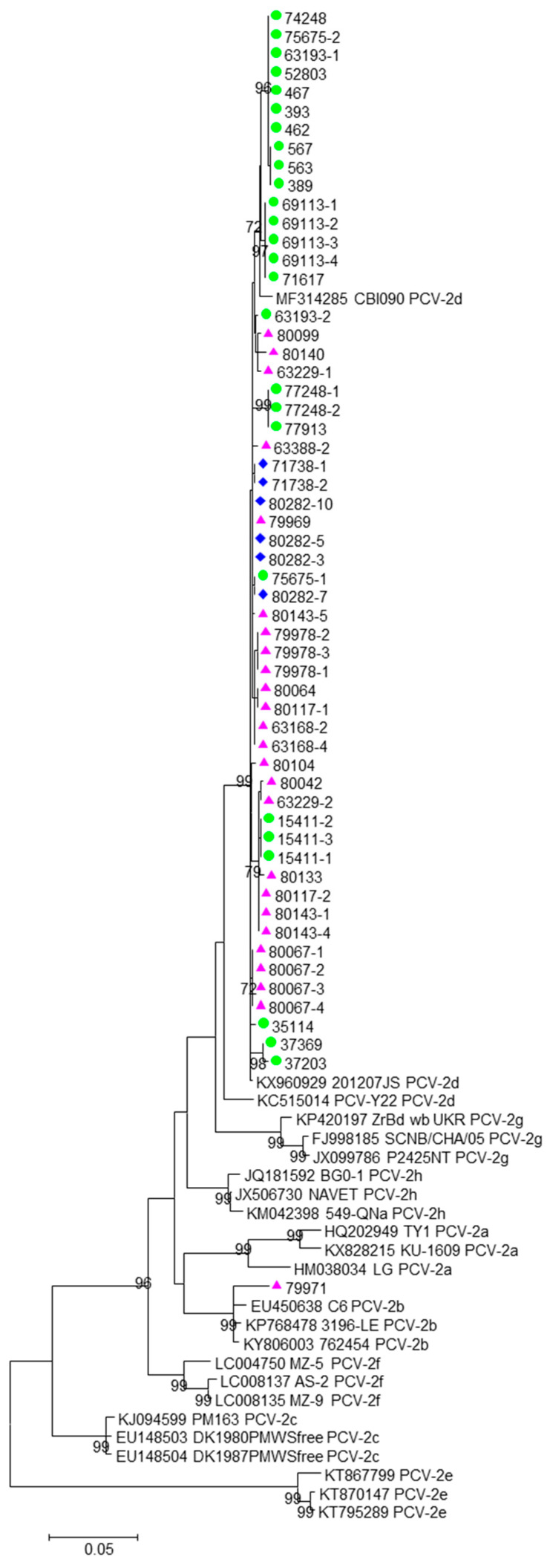
A maximum likelihood phylogenetic tree inferred from the first data set by the GTR+G+I model of nucleotide substitution. Isolates under study are indicated with different symbols. Red triangles: wild boar; green circles: domestic pigs; purple diamonds: free-ranging pigs. Bootstrap values < 70 are not shown. The scale bar indicates the number of substitutions per site.

The recombinant analysis performed using RDP4 showed no evidence of recombination between the sequences included in the datasets analyzed. The phylogenetic signals, depicted in Figure S1, evidenced sufficient phylogenetic information. The phylogenetic tree obtained from the first dataset is shown in Figure 1.

Fifty-six sequences clustered in the PCV2d genotype, and only one sequence (sample 79971, from a wild boar) clustered with the PCV2b genotype (Table S1, Figure 1). No strains belonging to the other genotypes were found.

**Figure 2:**
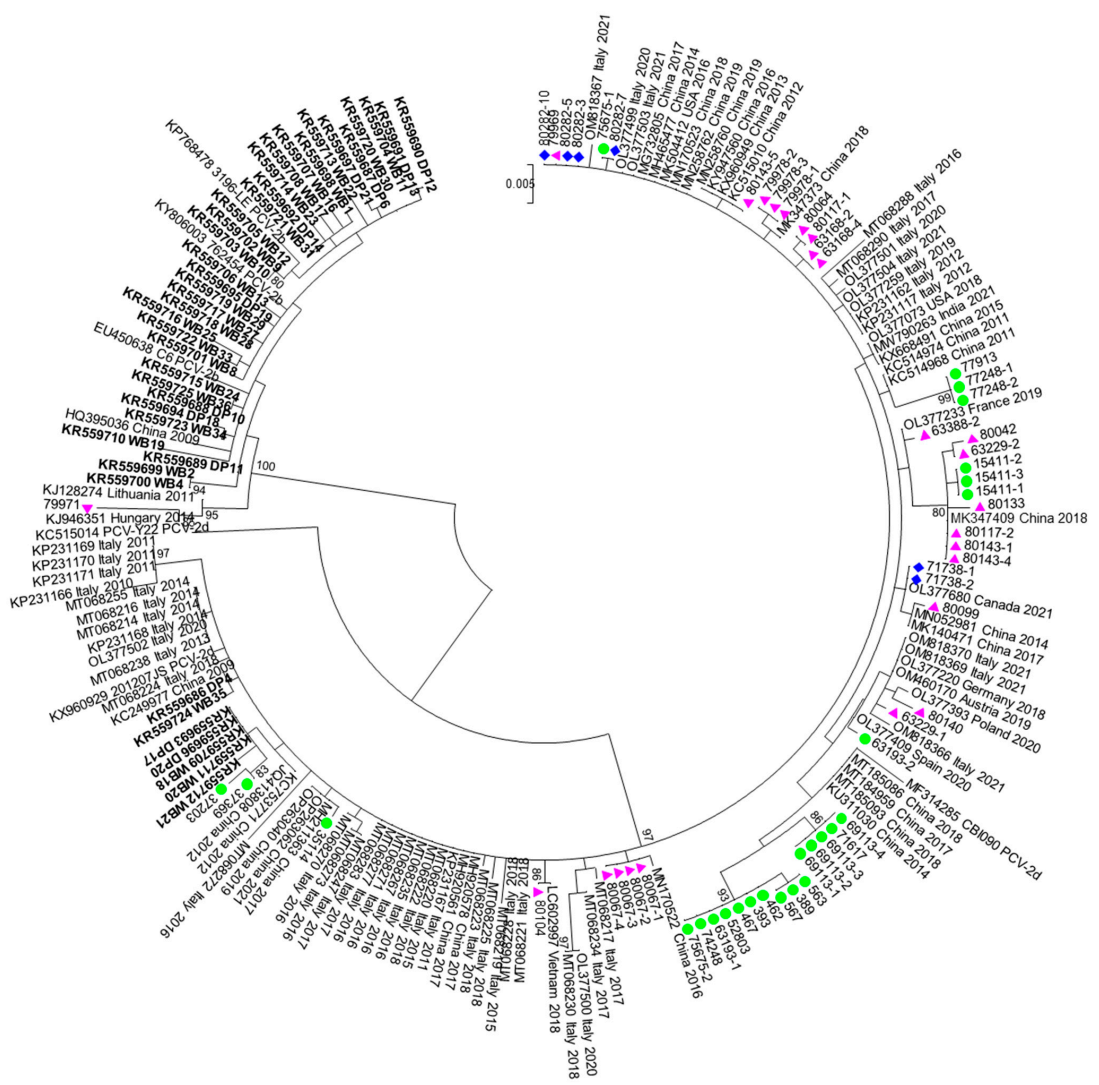
A maximum likelihood phylogenetic tree inferred from the second data set by the GTR+G+I model of nucleotide substitution. Isolates under study are indicated with different symbols. Red triangles: wild boar; green circles: domestic pigs; purple diamonds: free-ranging pigs. Sequences from our previous work are highlighted in bold. Bootstrap values < 70 are not shown. The scale bar indicates the number of substitutions per site.

The phylogenetic tree obtained from the second dataset is shown in Figure 2. It also allows the comparison of the sequences obtained in this work with those collected about 10 years ago in Sardinia in our previous study [38].

The majority (33/40) of the sequences collected in 2009–2013 belonged to PCV2b; on the contrary, almost all the sequences (56/57) obtained in this work were identified as PCV2d.

The Sardinian PCV2d strains clustered mostly with sequences from China but also Italy, Spain, Poland, France, and Germany showing 98.87–99.9% similarity with them. The strains 80282-3-5-10 along with 79969, coming from municipalities 150 km apart, showed 100% similarity with strains from China collected in 2014–2019, USA (2016), and Italy (2020–2021). The sequence 80104, 80143-1, 80143-5, and 71738 showed 100% similarity with strains from Vietnam (2018), China (2018), China (2012–2019), and Canada (2021), respectively.

A small cluster composed of three sequences collected from domestic pigs from two different Sardinian provinces (Table S1, Figure 2) is strongly supported (bootstrap 99%). Another cluster (bootstrap 80%) is composed of nine Sardinian sequences from domestic pigs and wild boars, coming from different municipalities in two Sardinian provinces, and they show the highest similarity with a Chinese strain isolated in 2018. Two neighbor clusters composed of strains from domestic pigs were statistically supported by high bootstrap values (86% and 93%) and are composed of sequences collected in three different Sardinian provinces (Table S1, Figure 2). Five out of seven PCV2d sequences from our previous work (DP17, DP20, WD18, WB20, WB21, collected in 2011–2012) clustered non-significantly with two new PCV2d sequences collected in 2022 from domestic pigs in the same municipality (Table S1, Figure 2), 30 and 60 km apart. Other non-significant clusters are composed of sequences from domestic and wild suids or consist of wild boar and free-ranging pigs PCV2 strains from different provinces.

**Figure 3:**
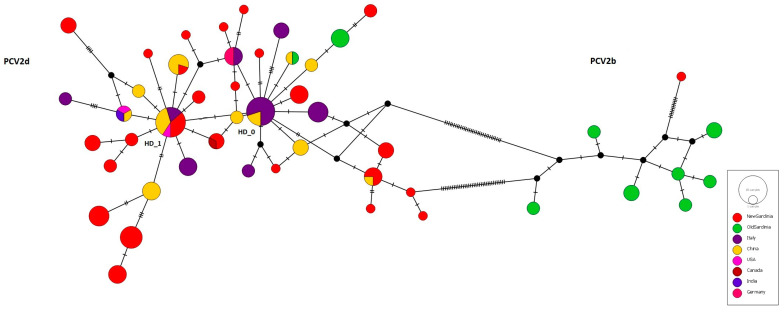
The haplotype network built on 138 ORF2 sequences extracted from the second dataset (Table S2). The geographical distribution of the haplotypes is indicated by different colours as shown in the legend. The size of the circles is proportional to the frequency of each haplotype. The number of mutations distinguishing the haplotypes is shown by hatch marks.

The network analysis, depicted In Figure 3, confirmed the findings of the phylogenetic tree. Sardinian haplotypes were closely related to a major haplotype composed of sequences from mainland Italy and China (HD_0), and several strains belong to the haplotype HD_1, which comprises sequences from mainland Italy, China, and the USA. Sardinian-specific haplotypes containing more than two sequences and separated by one up to six mutational steps from the two main haplotypes were also evidenced.

The amino acid alignment of the different haplotypes found in this study with the reference strains PCV2b and PCV2d [31] is shown in Figure S2. The sample 79971 belonging to the PCV2b genotype showed three amino acid substitutions (T131P, L185R, and M212L) compared to the reference strains (Figure S2a). The same substitutions were found in two sequences from Hungary (KJ946351, 2014) and Lithuania (KJ128274, 2011). Blast analysis showed that these sequences have the highest similarity (99.01% and 98.87%, respectively) with this strain. Instead, the alignment of the PCV2d sequences evidenced five substitutions (sample 77913: I22L and V193I; sample 80140: T131R and T217I; sample 63388: V101I) and four polymorphisms at position 169 (R/G/T/S) (Figure S2b). The amino acid replacements V193I was found in a single sequence from USA (APJ37574, 2016), T217I in the sequences UPQ64522 (France, 2020) and UPQ64565 (Vietnam, 2019), V101I in the sequences UJP85687 (China, 2018), and QBG82190 (China, 2015). The polymorphisms at position 169 were already described in several sequences in the GenBank database.

The amino acid replacements I22L and T131R are unique in the GenBank database. The amino acid 22 is part of a nuclear localization signal (NLS), and it is located in the N-terminus of the protein inside the capsid [48,49,50]. Instead, the amino acid 131 is located in the EF loop, which contributes to the capsid surface features [48]. Epitope analysis for the sequence containing T131R mutation identified two potential B cell epitopes ^129^FVRKANALTYDPYVNY^144^ and ^121^TAVILDDNFVRKANAL^136^, whereas no T cell epitopes were evidenced in position 131. Mutation analysis evidenced that T131R did not alter the secondary structure compared to the reference strains (PBD ID 3R0R:A and 3JCI:A). It was not possible to perform the same analysis for I22L because only reference sequences without the N-terminal portion were found in the PDB database.

## 4. Discussion

In the present study, we analyzed samples of domestic pigs, free-ranging pigs, and wild boar collected in Sardinia from 2020 to 2022 to detect the presence of the PCV2 genome. An update on the genetic variants circulating in the Italian island was provided following the last survey performed on samples collected between 2009 and 2013.

The overall PCV2 infection rate found in this work was 55.28%. Significantly lower values were found in domestic pigs (36.27%) compared to free-ranging pigs (87.3%) and wild boars (73.53%). These differences between infection rates of domestic and wild suids might reflect the different immunological responses of a vaccinated versus a naïve population and the application of biosecurity control measures in domestic pig farms. As previously stated, these data confirmed the role of wild boars as reservoirs of PCV2 in Sardinia and suggested that free-ranging pigs might be responsible for virus shedding between rural and sylvan environments [19,38,51]. Unfortunately, the limited number of free-ranging pig sequences obtained in this work prevents further conclusions.

While the knowledge about the PCV2 prevalence or detection rate in domestic pigs in Italy is limited, the presence of PCV2 in wild boars using molecular methods on serum and tissue samples has been carried out by previous studies [19,21,22,51]. Infection rates from Sardinian wild boars found in the present investigation were similar to what was already reported by our group 3 years ago (89.74%, 2018–2019 hunting season) and higher than those reported in Southern (Basilicata 27%, Campania 47.3%) and Northern Italy (Friuli Venezia Giulia and Veneto, 54.9%). However, infection rates reported in scientific literature are difficult to compare, and animal health status, animal age, samples matrix, and epidemiological situation may account for the differences among published studies. Particularly, the Sardinian epidemiological scenario is also characterized by the presence of numerous family-run farms with a limited number of animals that, in certain rural areas, were illegally reared under free-ranging conditions. This traditional practice, related to socio-economic and cultural aspects, sadly played a key role in the African Swine Fever Virus (ASFV) persistence in Sardinia. Nowadays, as a consequence of measures adopted in the framework of the ASFV eradication plan of the region Sardinia of 2015 and subsequent additions [52] and in consideration of increased awareness of the local population, the free-ranging pigs are considered a marginal phenomenon with sporadic sighting throughout the regional territory.

The phylogenetic analysis revealed that almost all the sequences (56 out of 57 strains) obtained in this study from samples collected between 2020 and 2022 belong to the PCV2d genotype. Only one sequence from a wild boar clustered with the PCV2b genotype.

The presence of several well-supported clusters of PCV2d strains suggested the occurrence of different virus introduction events. Moreover, these clusters included Sardinian strains as well as sequences from mainland Italy and other countries. As already reported, the importation of live animals from peninsular Italy and other European countries could explain the high similarity of virus strains circulating in Sardinia with those found in extremely distant geographical areas [8]. In the same way, no differences between the viral strains collected from domestic and wild suids could be evidenced, thus supporting the hypothesis that wild boar and free-ranging pigs may be responsible for the maintaining and spreading of the virus throughout the regional territory and the establishment of a recurrent viral flux at the domestic–wildlife interface. Our findings can also suggest, as it also appears in the network analysis, that viral strains introduced on the island of Sardinia are subjected to local evolution generating new unique variants (I22L and T131R). This is also confirmed by the presence of two Sardinian neighbour clusters composed only of domestic pig samples, which probably evolved from foreign strains and spread between Sardinian provinces through local animal exchanges. We have also evidenced that T131R mutation did not affect the spatial conformation of the virus, but the B cell epitopes were changed with respect to the reference strain.

The main finding of the present study was the evidence of the exclusive circulation of PCV2d genotype in Sardinia from 2020 to 2022, in contrast with the results of the previous study performed with sequences collected about 10 years ago, thus strongly suggesting the occurrence of a genetic shift from PCV2b to PCV2d.

A phylogenetic study by Franzo et al. [19], based on sequences collected up to 2018, reported that, differently from what was observed in other countries, a proper genotype replacement could not be stated in Italy. As highlighted in this paper, the Sardinian epidemiological scenario cannot be representative of the entire Italian pig industry, especially not of the major swine-producing region of Northern Italy. Nonetheless, Sardinian pig production is more similar to the farming method applied in certain areas of Southern and Central Italy characterized by small semi-intensive farms. The presence of ASFV in Sardinia from 1978 and the consequent control measures adopted could have influenced swine viruses’ evolution. We can speculate that the application of the ASFV eradication plan, which led to a drastic decrease in the number of domestic and free-ranging pigs, might have accelerated the replacement of PCV2b, particularly due to the reintroductions of animals, mainly infected with PCV2d. In addition to this, the insular nature, along with the animal movement ban, might have created favorable conditions for the evolution of new viral variants.

Recently, other studies performed in Italy on samples collected between 2018 and 2022 have reported that all but one of the sequences obtained belonged to PCV2d [19,22,53,54]. This might be indicative that, from 2018 onwards, the complete genotype shift has also occurred in mainland Italy. The authors would also like to stress the changed epidemiological situation in Sardinia in the last two years, which led to the softening of the national export restrictions towards their expected elimination in 2024. The renewal of bidirectional exchange of live pigs and pig products between Sardinia and the peninsula would possibly influence PCV2 epidemiology in Italy.

## Figures and Tables

**Table 1:** PCV2 infection rate of the samples analyzed in this study.

Sample	Domestic Pigs Infection Rate (%)	Free-Ranging Pigs Infection Rate (%)	Wild Boars Infection Rate (%)	Total Infection Rate (%)
Blood serum	24/89 (26.97) ^a^	/	26/44 (59.09) ^b^	50/133 (37.59)
Tissues	50/115 (43.48) ^a^	55/63 (87.30) ^b^	49/58 (84.48) ^b^	154/236 (65.24)
Total	74/204 (36.27) ^a^	55/63 (87.30) ^b^	75/102 (73.53) ^b^	204/369 (55.28%)

Chi-squared test (Χ^2^) was performed between infection rate values of domestic pigs, free-ranging pigs, and wild boards. Different superscripts within the rows indicate significant differences between the three groups (*p* < 0.05).

## Data Availability

Not applicable.

## Supplementary Materials

The following supporting information can be downloaded at: https://www.mdpi.com/article/10.3390/v15112157/s1, Figure S1. Phylogenetic signals of the data sets analyzed in this study; Figure S2. Amino acid alignment of Sardinian PCV2 sequences with the PCV2 reference strains; Table S1. List of PCV2 strains sequenced in this study, source, geographical origin, and GenBank accession number. (*n* = 57). Table S2. List of PCV2 ORF2 sequences used to calculate the haplotype network (*n* = 138), sample details, and GenBank accession number.
