# Supplementary material for: Evidence of Porcine Circovirus Type 2 (PCV2) Genetic Shift from PCV2b to PCV2d Genotype in Sardinia, Italy
Source: Viruses. 2023 Oct 26;15(11):2157. doi: 10.3390/v15112157 (PMC10674684; doi:10.3390/v15112157)
Supplement: Supplementary file 1 [file viruses-15-02157-s001.zip › viruses-2632091-supplementary/Figure S1.pptx]

## Slide 1
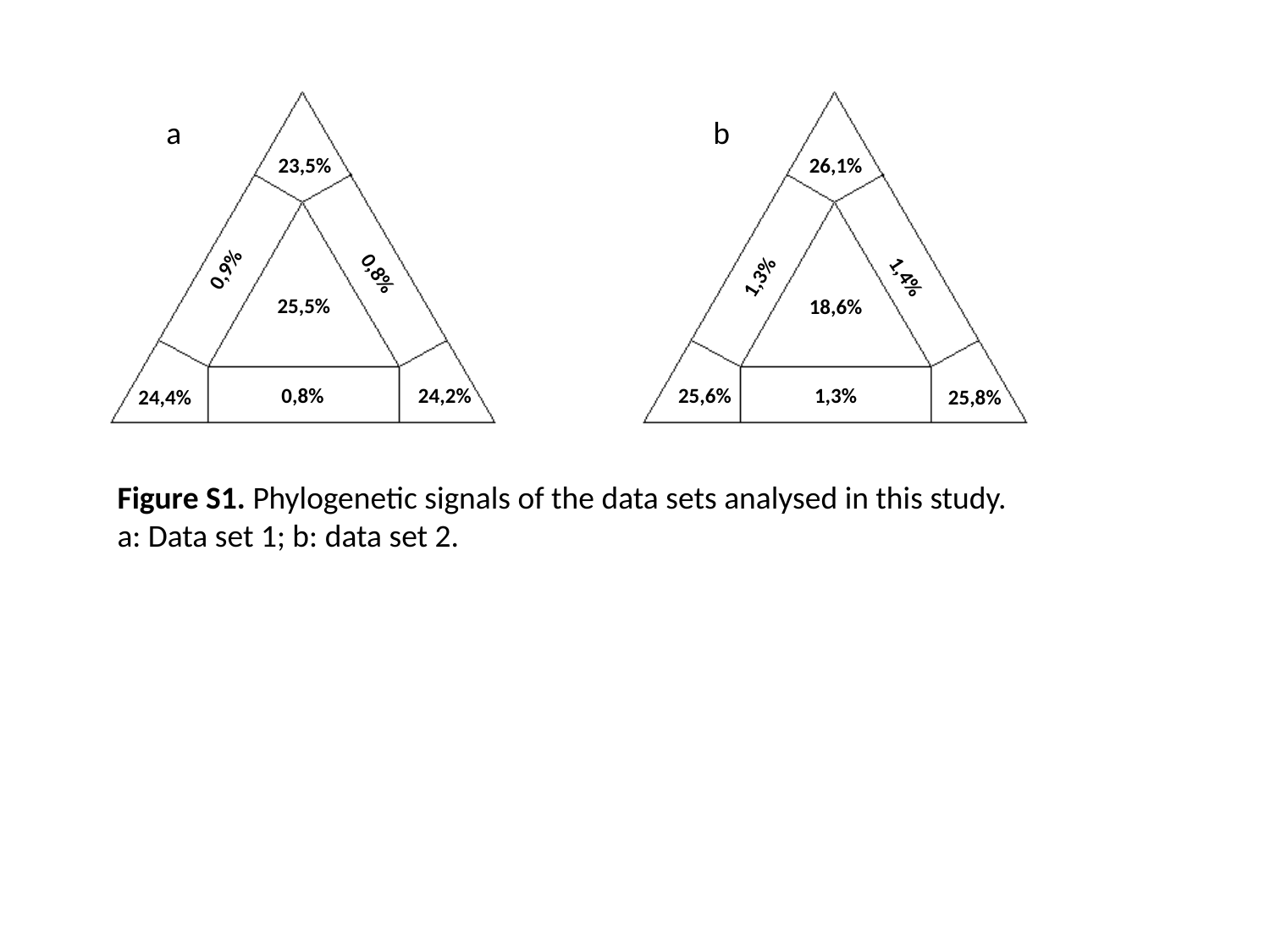

23,5%
0,9%
0,8%
25,5%
24,2%
0,8%
24,4%
26,1%
1,4%
1,3%
18,6%
25,6%
1,3%
25,8%
a
b
Figure S1. Phylogenetic signals of the data sets analysed in this study.
a: Data set 1; b: data set 2.
