# Supplementary material for: Evidence of Porcine Circovirus Type 2 (PCV2) Genetic Shift from PCV2b to PCV2d Genotype in Sardinia, Italy
Source: Viruses. 2023 Oct 26;15(11):2157. doi: 10.3390/v15112157 (PMC10674684; doi:10.3390/v15112157)
Supplement: Supplementary file 1 [file viruses-15-02157-s001.zip › viruses-2632091-supplementary/Figure S2.pdf]

```
                                   10        20        30        40        50        60        70        80        90       100
                           ....|....|....|....|....|....|....|....|....|....|....|....|....|....|....|....|....|....|....|....|
EU450638 C6 PCV-2b         MTYPRRRYRRRRHRPRSHLGQILRRRPWLVHPRHRYRWRRKNGIFNTRLSRTFGYTIKRTTVRTPSWAVDMMRFNINDFLPPGGGSNPRSVPFEYYRIRK
KP768478 3196-LE PCV-2b    ..............................................................K.....................................
KY806003 762454 PCV-2b     ................................H.............................K.....................................
79971                      ..............................................................K.....................................

                                  110       120       130       140       150       160       170       180       190       200
                           ....|....|....|....|....|....|....|....|....|....|....|....|....|....|....|....|....|....|....|....|
EU450638 C6 PCV-2b         VKVEFWPCSPITQGDRGVGSSAVILDDNFVTKATALTYDPYVNYSSRHTITQPFSYHSRYFTPKPVLDSTIDYFQPNNKRNQLWLRLQTAGNVDHVGLGT
KP768478 3196-LE PCV-2b    ....................................................................................................
KY806003 762454 PCV-2b     ....................................................................................................
79971                      ..............................P.....................................................M...............

                                  210       220       230
                           ....|....|....|....|....|....|....
EU450638 C6 PCV-2b         AFENSIYDQEYNIRVTMYVQFREFNLKDPPLNP*
KP768478 3196-LE PCV-2b    .................................*
KY806003 762454 PCV-2b     ...............................K.*
79971                      ................L................*
```

Figure S2a. Amino acid alignment of the sample 79971 with the PCV2b reference strains [31].

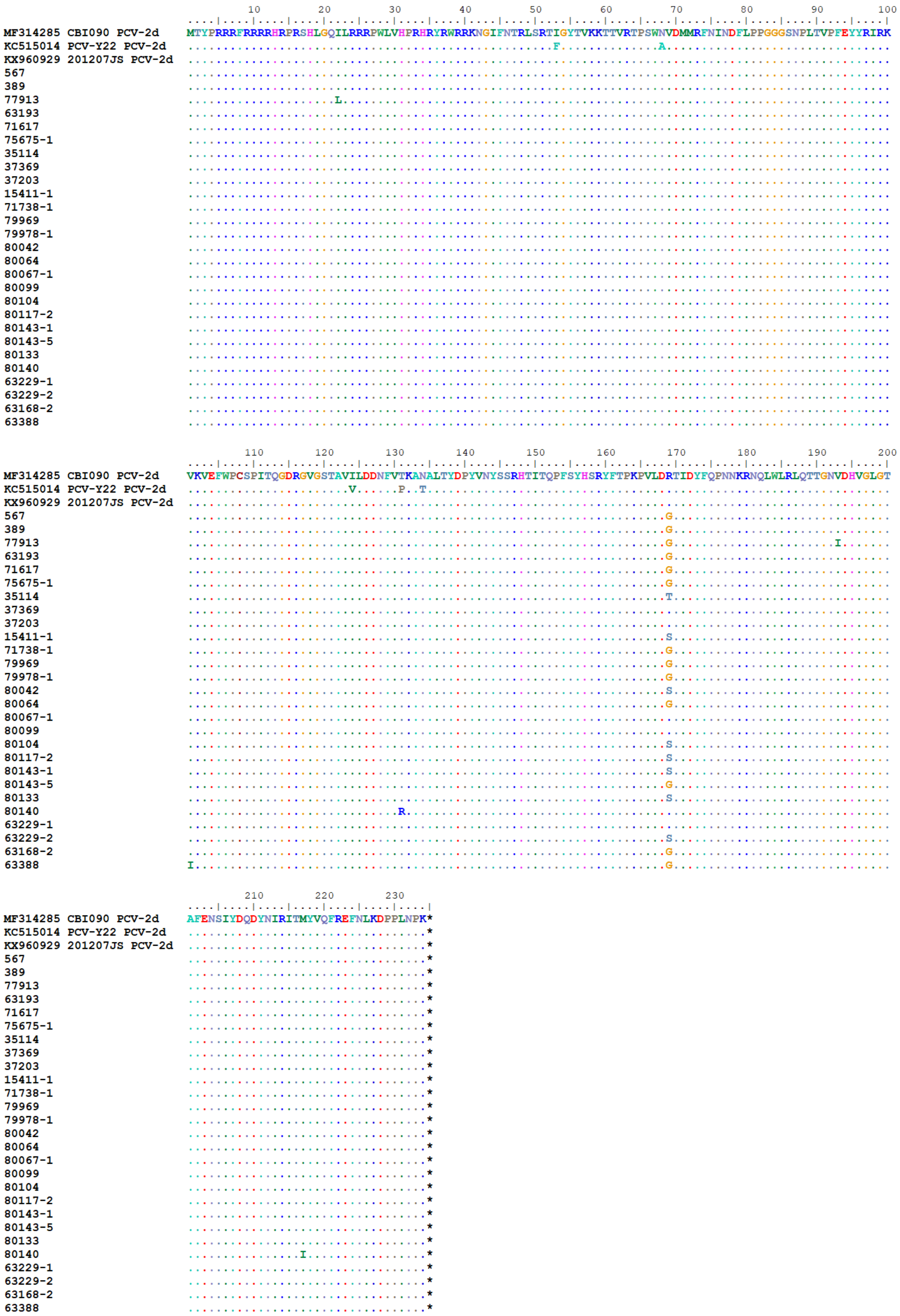

Figure S2b. Amino acid alignment of the different haplotypes evidenced in this study with PCV2d reference strains [31].
