# Supplementary material for: Evidence of Porcine Circovirus Type 2 (PCV2) Genetic Shift from PCV2b to PCV2d Genotype in Sardinia, Italy
Source: Viruses. 2023 Oct 26;15(11):2157. doi: 10.3390/v15112157 (PMC10674684; doi:10.3390/v15112157)
Supplement: Supplementary file 1 [file viruses-15-02157-s001.zip › viruses-2632091-supplementary/Table S1.pdf]

Table S1. List of PCV2 strains sequenced in this study, source, geographical origin and GenBank accession number. (n=57)

Strain	Source	Municipality	Province	GenBank Accession Number
**567**	**Ln mesenteric**	San Gavino Monreale	Sud Sardegna	OR19918
**563**	**Lung**	San Gavino Monreale	Sud Sardegna	OR19919
**462**	**Ln mediastinal**	San Gavino Monreale	Sud Sardegna	OR19920
**389**	**Spleen**	San Gavino Monreale	Sud Sardegna	OR19921
**393**	**Kidney**	San Gavino Monreale	Sud Sardegna	OR19922
**467**	**Lung**	San Gavino Monreale	Sud Sardegna	OR19923
**77913**	**Placenta**	Usellus	Oristano	OR19924
**77248-1**	**Foetus brain**	Gonnosfanadiga	Sud Sardegna	OR19925
**77248-2**	**Placenta**	Gonnosfanadiga	Sud Sardegna	OR19926
**69113-1**	**Foetus spleen**	Sanluri	Sud Sardegna	OR19927
**69113-2**	**Foetus brain**	Sanluri	Sud Sardegna	OR19928
**69113-3**	**Foetus spleen**	Sanluri	Sud Sardegna	OR19929
**69113-4**	**Foetus brain**	Sanluri	Sud Sardegna	OR19930
**52803**	**Lung**	Villamassargia	Sud Sardegna	OR19931
**63193-1**	**Heart**	Orosei	Nuoro	OR19932
**63193-2**	**Lung**	Orosei	Nuoro	OR19933
**71617**	**Heart**	Talana	Nuoro	OR19934
**74248**	**Foetus brain**	Pattada	Sassari	OR19935
**75675-1**	**Foetus spleen**	Irgoli	Nuoro	OR19936
**75675-2**	**Foetus spleen**	Irgoli	Nuoro	OR19937
**35114**	**Serum**	Austis	Nuoro	OR19938
**37369**	**Serum**	Berchidda	Hap_22	OR19939
**37203**	**Serum**	Berchidda	Hap_22	OR19940
**15411-1**	**Foetus spleen**	Sassari	Hap_24	OR19941
**15411-2**	**Foetus spleen**	Sassari	Hap_24	OR19942
**15411-3**	**Foetus brain**	Sassari	Hap_24	OR19943
**80282-3**	**spleen**	Talana	Nuoro	OR19944
**80282-5**	**spleen**	Talana	Nuoro	OR19945
**80282-7**	**spleen**	Talana	Nuoro	OR19946
**80282-10**	**spleen**	Talana	Nuoro	OR19947
**71738-1**	**spleen**	Urzulei	Nuoro	OR19948
**71738-2**	**spleen**	Urzulei	Nuoro	OR19949
**79969**	**spleen**	Monti	Sassari	OR19950
**79971**	**spleen**	Ittireddu	Sassari	OR19951
**79978-1**	**spleen**	Nughedu San Nicolò	Sassari	OR19952
**79978-2**	**spleen**	Nughedu San Nicolò	Sassari	OR19953
**79978-3**	**spleen**	Nughedu San Nicolò	Sassari	OR19954
**80042**	**spleen**	Bono	Sassari	OR19955
**80064**	**spleen**	Bono	Sassari	OR19956
**80067-1**	**spleen**	Benetutti	Sassari	OR19957
**80067-2**	**spleen**	Benetutti	Sassari	OR19958
**80067-3**	**spleen**	Benetutti	Sassari	OR19959
**80067-4**	**spleen**	Benetutti	Sassari	OR19960
**80099**	**spleen**	Benetutti	Sassari	OR19961
**80104**	**spleen**	Alà dei Sardi	Sassari	OR19962
**80117-1**	**spleen**	Bonorva	Sassari	OR19963
**80117-2**	**spleen**	Bonorva	Sassari	OR19964
**80143-1**	**spleen**	Illorai	Sassari	OR19965
**80143-4**	**spleen**	Illorai	Sassari	OR19966
**80143-5**	**spleen**	Illorai	Sassari	OR19967
**80133**	**spleen**	Arzachena	Sassari	OR19968
**80140**	**spleen**	Pattada	Sassari	OR19969
**63229-1**	**spleen**	Pattada	Sassari	OR19970
**63229-2**	**spleen**	Pattada	Sassari	OR19971
**63168-2**	**spleen**	Oschiri	Sassari	OR19972

**63168-4**	**spleen**	Oschiri	Sassari	OR19973
**63388**	**spleen**	Tula	Sassari	OR19974
