# Supplementary material for: Evidence of Porcine Circovirus Type 2 (PCV2) Genetic Shift from PCV2b to PCV2d Genotype in Sardinia, Italy
Source: Viruses. 2023 Oct 26;15(11):2157. doi: 10.3390/v15112157 (PMC10674684; doi:10.3390/v15112157)
Supplement: Supplementary file 1 [file viruses-15-02157-s001.zip › viruses-2632091-supplementary/Table S2.pdf]

Table S2. List of PCV2 ORF2 sequences used to calculate the haplotype network (n=138), samples details and GenBank accession number.

Haplotype in this study	Samples (n)	Host	Country of origin	Year of collection	Genotype	GenBank Accession Number
Hap_1	1	Wild boar	Italy (Sardinia)	2021	PCV2b	OR19951
Hap_2	3	Wild boar	Italy (Sardinia)	2021	PCV2d	OR19952
		Wild boar	Italy (Sardinia)	2021	PCV2d	OR19953
		Wild boar	Italy (Sardinia)	2021	PCV2d	OR19954
Hap_3	1	Wild boar	Italy (Sardinia)	2021	PCV2d	OR19955
Hap_4	4	Wild boar	Italy (Sardinia)	2021	PCV2d	OR19957
		Wild boar	Italy (Sardinia)	2021	PCV2d	OR19958
		Wild boar	Italy (Sardinia)	2021	PCV2d	OR19959
		Wild boar	Italy (Sardinia)	2021	PCV2d	OR19960
Hap_5	1	Wild boar	Italy (Sardinia)	2021	PCV2d	OR19961
Hap_6	1	Wild boar	Italy (Sardinia)	2021	PCV2d	OR19962
Hap_7	2	Wild boar	Italy (Sardinia)	2021	PCV2d	OR19963
		Wild boar	Italy (Sardinia)	2021	PCV2d	OR19956
Hap_8	4	Wild boar	Italy (Sardinia)	2021	PCV2d	OR19964
		Wild boar	Italy (Sardinia)	2021	PCV2d	OR19965
		Wild boar	Italy (Sardinia)	2021	PCV2d	OR19966
		Domestic pig	China	2018	PCV2d	MK347409
Hap_9	5	Wild boar	Italy (Sardinia)	2021	PCV2d	OR19967
		Domestic pig	China	2019	PCV2d	MN258760
		Domestic pig	China	2016	PCV2d	KY947560
		Domestic pig	China	2013	PCV2d	KX960949
		Domestic pig	China	2012	PCV2d	KC515010
Hap_10	1	Wild boar	Italy (Sardinia)	2021	PCV2d	OR19968
Hap_11	1	Wild boar	Italy (Sardinia)	2021	PCV2d	OR19969
Hap_12	11	Domestic pig	China	2019	PCV2d	MN258762
		Domestic pig	China	2018	PCV2d	MN170523
		Domestic pig	USA	2016	PCV2d	MK504412
		Domestic pig	China	2014	PCV2d	MH465477
		Domestic pig	China	2017	PCV2d	MG732805
		Domestic pig	Italy	2021	PCV2d	OL377503
		Domestic pig	Italy	2020	PCV2d	OL377499
		Free ranging pi	Italy (Sardinia)	2021	PCV2d	OR19944
		Free ranging pi	Italy (Sardinia)	2021	PCV2d	OR19945
		Free ranging pi	Italy (Sardinia)	2021	PCV2d	OR19947
		Wild boar	Italy (Sardinia)	2021	PCV2d	OR19950
Hap_13	1	Wild boar	Italy (Sardinia)	2022	PCV2d	OR19970
Hap_14	1	Wild boar	Italy (Sardinia)	2022	PCV2d	OR19971
Hap_15	2	Wild boar	Italy (Sardinia)	2022	PCV2d	OR19972
		Wild boar	Italy (Sardinia)	2022	PCV2d	OR19973
Hap_16	1	Wild boar	Italy (Sardinia)	2022	PCV2d	OR19974
Hap_17	3	Domestic pig	Italy (Sardinia)	2021	PCV2d	OR19924
		Domestic pig	Italy (Sardinia)	2021	PCV2d	OR19925
		Domestic pig	Italy (Sardinia)	2021	PCV2d	OR19926
Hap_18	5	Domestic pig	Italy (Sardinia)	2021	PCV2d	OR19927
		Domestic pig	Italy (Sardinia)	2021	PCV2d	OR19928
		Domestic pig	Italy (Sardinia)	2021	PCV2d	OR19929
		Domestic pig	Italy (Sardinia)	2021	PCV2d	OR19930
		Domestic pig	Italy (Sardinia)	2022	PCV2d	OR19934
Hap_19	2	Domestic pig	Italy (Sardinia)	2022	PCV2d	OR19936
		Free ranging pig	Italy (Sardinia)	2022	PCV2d	OR19946
Hap_20	6	Domestic pig	Italy (Sardinia)	2020	PCV2d	OR19922
		Domestic pig	Italy (Sardinia)	2020	PCV2d	OR19931

		Domestic pig	Italy (Sardinia)	2020	PCV2d	OR19932
		Domestic pig	Italy (Sardinia)	2020	PCV2d	OR19932
		Domestic pig	Italy (Sardinia)	2020	PCV2d	OR19935
		Domestic pig	Italy (Sardinia)	2020	PCV2d	OR19937
Hap_21	1	Domestic pig	Italy (Sardinia)	2022	PCV2d	OR19933
Hap_22	2	Domestic pig	Italy (Sardinia)	2022	PCV2d	OR19939
		Domestic pig	Italy (Sardinia)	2022	PCV2d	OR19940
Hap_23	1	Domestic pig	Italy (Sardinia)	2022	PCV2d	OR19938
Hap_24	3	Domestic pig	Italy (Sardinia)	2023	PCV2d	OR19941
		Domestic pig	Italy (Sardinia)	2023	PCV2d	OR19942
		Domestic pig	Italy (Sardinia)	2023	PCV2d	OR19943
Hap_25	4	Domestic pig	Italy (Sardinia)	2020	PCV2d	OR19918
		Domestic pig	Italy (Sardinia)	2020	PCV2d	OR19919
		Domestic pig	Italy (Sardinia)	2020	PCV2d	OR19920
		Domestic pig	Italy (Sardinia)	2020	PCV2d	OR19921
Hap_26	3	Domestic pig	Canada	2021	PCV2d	OL377680
		Free ranging pig	Italy (Sardinia)	2022	PCV2d	OR19948
		Free ranging pig	Italy (Sardinia)	2022	PCV2d	OR19949
Hap_27	3	Wild boar	Italy (Sardinia)	2011	PCV2b	KR559719
		Wild boar	Italy (Sardinia)	2011	PCV2b	KR559718
		Wild boar	Italy (Sardinia)	2011	PCV2b	KR559717
Hap_28	4	Domestic pig	Italy (Sardinia)	2012	PCV2d	KR559696
		Domestic pig	Italy (Sardinia)	2012	PCV2d	KR559693
		Wild boar	Italy (Sardinia)	2011	PCV2d	KR559711
		Wild boar	Italy (Sardinia)	2011	PCV2d	KR559709
Hap_29	3	Wild boar	Italy (Sardinia)	2010	PCV2b	KR559705
		Wild boar	Italy (Sardinia)	2010	PCV2b	KR559703
		Wild boar	Italy (Sardinia)	2010	PCV2b	KR559702
Hap_30	3	Domestic pig	China	2011	PCV2d	KX668491
		Domestic pig	USA	2018	PCV2d	OL377073
		Domestic pig	India	2021	PCV2d	MW790263
Hap_31	2	Domestic pig	China	2011	PCV2d	KC514974
		Domestic pig	China	2011	PCV2d	KC514968
Hap_32	4	Domestic pig	Austria	2019	PCV2d	OM460170
		Domestic pig	Germany	2018	PCV2d	OL377220
		Wild boar	Italy	2021	PCV2d	OM818370
		Wild boar	Italy	2021	PCV2d	OM818369
Hap_33	4	Domestic pig	China	2014	PCV2d	KU311030
		Domestic pig	China	2018	PCV2d	MT185093
		Domestic pig	China	2018	PCV2d	MT185086
		Domestic pig	China	2017	PCV2d	MT184959
Hap_34	10	Domestic pig	China	2017	PCV2d	MH920578
		Domestic pig	China	2017	PCV2d	MH920561
		Domestic pig	Italy	2011	PCV2d	KP231167
		Domestic pig	Italy	2015	PCV2d	MT068220
		Domestic pig	Italy	2018	PCV2d	MT068222
		Domestic pig	Italy	2016	PCV2d	MT068235
		Domestic pig	Italy	2016	PCV2d	MT068267
		Domestic pig	Italy	2016	PCV2d	MT068271
		Domestic pig	Italy	2016	PCV2d	MT068277
		Wild boar	Italy	2017	PCV2d	MT068283
Hap_35	3	Domestic pig	China	2017	PCV2d	MH211363
		Domestic pig	China	2021	PCV2d	OP263062
		Domestic pig	China	2019	PCV2d	OP263040
Hap_36	4	Domestic pig	Italy	2021	PCV2d	OL377504
		Domestic pig	Italy	2019	PCV2d	OL377259
		Domestic pig	Italy	2012	PCV2d	KP231162

		Domestic pig	Italy	2012	PCV2d	KP231117
Hap_37	5	Domestic pig	Italy	2014	PCV2d	MT068214
		Domestic pig	Italy	2014	PCV2d	MT068216
		Domestic pig	Italy	2014	PCV2d	MT068255
		Domestic pig	Italy	2014	PCV2d	KP231168
		Domestic pig	Italy	2020	PCV2d	OL377502
Hap_38	3	Domestic pig	Italy	2011	PCV2d	KP231171
		Domestic pig	Italy	2011	PCV2d	KP231170
		Domestic pig	Italy	2011	PCV2d	KP231169
Hap_39	2	Wild boar	Italy	2011	PCV2b	KR559725
		Wild boar	Italy	2011	PCV2b	KR559715
Hap_40	2	Domestic pig	Italy	2012	PCV2b	KR559692
		Wild boar	Italy	2011	PCV2b	KR559721
Hap_41	2	Wild boar	Italy	2011	PCV2b	KR559720
Hap_41		Wild boar	Italy	2010	PCV2b	KR559704
Hap_42	2	Wild boar	Italy	2009	PCV2b	KR559700
		Wild boar	Italy	2009	PCV2b	KR559699
Hap_43	2	Domestic pig	Italy	2012	PCV2b	KR559691
		Domestic pig	Italy	2012	PCV2b	KR559690
Hap_44	2	Domestic pig	Italy	2012	PCV2d	KR559686
		Domestic pig	China	2009	PCV2d	KC249977
Hap_45	2	Domestic pig	China	2014	PCV2d	MN052981
		Domestic pig	China	2017	PCV2d	MK140471
Hap_46	2	Domestic pig	China	2012	PCV2d	KC753771
		Domestic pig	China	2012	PCV2d	JQ413808
Hap_47	2	Domestic pig	Italy	2020	PCV2d	OL377500
		Domestic pig	Italy	2018	PCV2d	MT068230
Hap_48	2	Domestic pig	Italy	2017	PCV2d	MT068247
		Domestic pig	Italy	2016	PCV2d	MT068273
